# Runners' metabolomic changes following marathon

**DOI:** 10.1186/s12986-020-00436-0

**Published:** 2020-03-13

**Authors:** Rengfei Shi, Jin Zhang, Biqing Fang, Xiangyang Tian, Yu Feng, Zepeng Cheng, Zhongyu Fu, Jingjing Zhang, Jiaxi Wu

**Affiliations:** 1 School of Kinesiology, Shanghai University of Sport, 188 Hengren Road, Yangpu District, Shanghai, 200438 China; 2 Central Laboratories, Xuhui Central Hospital, Shanghai Clinical Research Center, Chinese Academy of Sciences, 966 Huaihai Middle Road, Shanghai, 200031 China

**Keywords:** Metabolomics, Marathon, Exercise, Serum

## Abstract

**Introduction:**

Marathon, as a long-distance aerobic exercise, has become a fashionable or popular sport. However, little is known about the holistic metabolic changes occurring within the serum metabolome of athletes after the completion of a marathon.

**Objectives:**

The goal of current study was to have an in-depth understanding of the impact of marathon on human metabolomics as well as the relationships among a variety of metabolites.

**Methods:**

The 20 studied subjects were all adult males who participated in a marathon. The serum samples of these participants were collected before and after the marathon and the biochemical metabolites in the serum were identified by an untargeted two-dimensional gas chromatography time-of-flight mass spectrometry.

**Results:**

All participants completed the marathon within 3 h. Compared to those before exercise, serum urea and creatine kinase, as well as cortisol, elevated significantly (*p* < 0.05), whereas testosterone decreased significantly (*p* < 0.01). Metabolomic analysis showed that, compared to those before the competition, metabolites pyruvic acid, glyceric acid, malic acid, cis-aconitic acid, galacturonic acid, methyl fumaric acid, maltotriose, and others increased significantly after the competition (*p* < 0.05), but glucosamine and O-succinyl-L-homoserine decreased significantly (*p* < 0.05). Amino acid indexes, such as alanine, L-tyrosine and phenylalanine, increased significantly after exercise compared with those before exercise (*p* < 0.05), whereas serine, valine and asparagine decreased significantly (*p* < 0.05). Lipid metabolism indexes, glycerol, glyceric acid, octanoic acid, and quinic acid increased significantly (*p* < 0.05). Theophylline, xanthine and other indicators of caffeine metabolism increased significantly (*p* < 0.05). Furthermore, marathon performance, fat percentage, VO_2_max, and hemoglobin were correlated with the serum metabonomic indicators, so were serum testosterone and cortisol.

**Conclusion:**

These results illustrate that the metabolism of glucose and lipid of the athletes was enhanced following the marathon match. In addition, the metabolism of glucosamine was decreased and the metabolism of caffeine was increased. Our data provide new insights for marathon performance and nutritional status.

## Introduction

Marathon, as one of the most important events in Olympic Games, fully embodies the concept of the Olympic Games motto: ‘Faster, Higher, Stronger’. In order to achieve excellent performance, runners make efforts in exercise training. Usually, after runners complete a full marathon match, their skeletal muscle, myocardium, liver, and other tissues are presented with an elevated free radical production, lipid peroxidation and disordered ion and energy metabolism, which lead to exercise-induced fatigue [1–3].

The growing popularity of marathon is an intriguing phenomenon. However, in recent years, unreasonable exercise has led to such health problems as system injury, hyponatremia, abnormal liver and kidney function, gastrointestinal dysfunction, and even sudden death, which have cast an unpleasant shadow on this sport [1, 4]. Much attention has been drawn to the promotion of physical exercise in order to prevent the associated diseases. The increased public interest in long-distance running has triggered an increased interest among scientists in investigating changes related to the performance of the sport. So far, numerous studies have been conducted on marathon runners; the studied aspects include the factors that influence marathon performance [5], economy of used energy [6] and relationship between running speed and VO_2_max [7]. However, as a mass sport, how to evaluate its fitness value remains to be determined. Mathews et al. [8] reported that in the years between 2000 and 2009, there were 28 fatal cases during or within 24 h after a marathon run in the USA.

Previous studies demonstrated that hematological and biochemical markers changed immediately following the sport and returned to baseline levels after 2–7 days of recovery in the amateur half-marathon runners [1]. Several hormonal markers, such as insulin, leptin and adiponectin, showed unique patterns after a marathon running [9].

The ability to supply energy by aerobic metabolism is a key factor in success in long-distance events. The coordinated energy supply provided by carbohydrate and fat metabolism, is particularly critical for achieving ideal marathon results [10]. The most commonly used blood indexes that describe health risk in marathon runners during performance and after-match recovery, are those that indicate liver damage, such as aspartate aminotransferase (AST), alanine aminotransferase (ALT) and lactate dehydrogenase (LDH), and cardiac or skeletal muscles damage, such as LDH, creatine kinase (CK) and C-reactive protein (CRP) [11]. Despite numerous studies, there is no consensus on whether such exertion is beneficial to health or which metabolic indicators change during the exercise. Several investigators have analyzed the physiologic and energetic requirements of endurance running. Others have reported that marathon increases bone formation rate in runners. A previous research in the rats observed that endurance training elevated the rate of tricarboxylic acid (TCA) cycle and antioxidant activity [12].

Recently, some researchers compared the changes of metabolic indicators between marathon runners and non-exercising people. But they only focused on the changes of some routine biochemical indicators in serum and did not investigate the more accurate metabolic indicators. Neither did they study the metabolomics differences before and after a marathon.

Emerging metabolite profiling technologies involve the analysis of all low molecular weight metabolites, in a quantitative manner, of a whole organism’s metabolic status within a defined time range under specific conditions [13, 14]. Metabolomics may have the potential for the application in the field of disease diagnosis and in the systematic view of the metabolic response to exercise stress [15]. The differences in metabolic indicators between pre- and post-marathon match might offer a clue. Here, we developed an untargeted liquid chromatography and mass spectrometry (LC/MS)-based metabolomics platform with high specificity for analyte identification and applied it to a series of blood samples obtained from the runners before and after marathon match. We then analyzed whether metabolites altered in response to exercise were correlated with critical fitness-related parameters.

## Materials and methods

### Participants

Twenty males volunteered for the study (age: 29.42 ± 4.51 years, height: 170.58 ± 8.23 cm, body weight: 59.10 ± 8.39 kg, body fat percentage: 10.45 ± 5.88). All the subjects were experienced amateur marathon runners who signed an informed consent form prior to the study. All participants were free from any health problems that would affect physical performance or put them at risk. The study was approved by the Ethics Committee of Shanghai University of Sport, China.

### Clinical samples

Blood samples were collected at the antebrachial vein of the fasting marathon amateur athletes between 8 am and 10 am the day preceding the race and within 1 h after completing the Shanghai International Marathon. The samples were collected in standard 5 mL vacutainer vials (Wenzhou Gaode Medical Instruments Co.,Ltd., #B1177696), placed on ice and transported to the Laboratory (Sports Molecular Biology Laboratory Center, Shanghai University of Sport) for immediate processing. Some of the blood samples were used for the analysis of serum biochemical indicators whereas the others, metabolomic indicators. The blood was allowed to clot for 30 min and centrifuged at 3000×g for 10 min. The supernatant (serum) was then extracted and immediately frozen (− 80 °C) until metabolomics analyses commenced.

### Biochemical analysis of serum samples

Total serum CK was measured by enzyme-coupled assay and its isoform CK-MB by immunosuppression assay (CK, #OSR6279; CK-MB, #OSR61155, Beckman Coulter Laboratory Systems Co.). Cortisol and testosterone were measured with Electrochemiluminescence Detection(testosterone, #05200067; Cortisol, #06687733, Roche Diagnostics GmbH). CRP was measured with immunoturbidimetry assay (CRP, #OSR6199 Beckman Coulter Laboratory Systems Co.). Homocysteine was determined by the enzymatic cycling method.

### Body composition detection and VO_2_max test

Body weight and height were measured using an ultrasound height and weight measuring instrument (HY-STL200, China). Fat mass and bone density were measured using a dual energy X-ray absorptiometry (GE Healthcare, Milwaukie, WI, USA). Body mass index (BMI) was calculated as body mass divided by squared height. VO_2_max tests were started at an initial velocity of 8 km/hr. (gradient = 1% throughout the test) proceeded with an increased speed of 0.5 km/hr. every 30 s until volitional exhaustion. Respiratory gas was determined using a portable metabolic analyzer (K5, Cosmed Srl, Rome, Italy), and VO_2_max was determined based on the highest 60-s average (e.g. average of two highest consecutive 30-s epochs) [16].

### Metabolites extraction

Samples were thawed on ice. 100 μL of sample was placed in an EP tube, extracted with 400 μL of extraction solvent (V methanol: V acetonitrile = 1:1) containing 2 μg/mL of internal standard, vortexed for 30 s, ultrasound-treated for 10 min (in ice water), incubated for 1 h at − 20 °C to precipitate proteins, and centrifuged at 13800 g for 15 min at 4 °C. The supernatant (425 μL) was transferred into EP tubes and the extracts were dries in a vacuum concentrator without heating.100 μL extraction solvent (V acetonitrile: V water = 1:1) was added for reconstitution. The reconstituted sample was vortex 30s and sonicated for 10 min in 4 °C water bath, centrifuged for 15 min at 13800 g at 4 °C. The supernatant (60 μL) was transferred into a fresh 2 mL LC/MS glass vial.10 μL from each sample was pooled as the QC sample. 60 μL supernatant of each sample was taken for the UHPLC-QTOF-MS analysis.

### LC-MS/MS analysis

The analysis of LC-MS/MS was performed using an UHPLC system (Agilent Technologies) with an UPLC BEH amide column (1.7 μm 2.1 mm*100 mm, Waters) coupled to Triple TOF 6600 (Q-TOF, AB Sciex). The fluid-phase that was consisted of 25 mM NH4Ac and 25 mM NH4OH in water (pH = 9.75) (A) and acetonitrile (B), was performed according to the following gradient elution method: 0 min(95% B); 0.5 min(95% B); 7 min(65% B); 8 min(40% B); 9 min(40% B); 9.1 min(95% B); 12 min(95% B), delivered at 0.5 mL/min, 2 μL of injection. The triple TOF mass spectrometer was applied for its ability to acquire MS/MS spectra on an information-dependent basis (IDA) during a LC/MS experiment. The acquisition software (Analyst TF 1.7, AB Sciex) continuously measures the full scan survey MS data as it collected and triggered the acquisition of MS/MS spectra that depend on preselected criteria. 12 precursor ions whose intensity greater than 100 were chosen for fragmentation at collision energy (CE) of 30 V (15 MS/MS events with product ion accumulation time of 50 msec each) in each cycle. Then, we set ESI source conditions: ion source gas 1 as 60 Psi, ion source gas 2 as 60 Psi, curtain gas as 35 Psi, source temperature 650 °C, Ion Spray Voltage Floating (ISVF) at 5000 V or − 4000 V in positive or negative modes, respectively [17, 18].

### Statistical analyses

The data matrix was center-formatted (mean-centered scaling) and Pareto-scaled prior to being imported into the SIMCA-P + 14.1 software package (Umetrics, Umea, Sweden). The multivariate data analysis (MVDA) included principal components analysis (PCA) and orthogonal partial least squared-discriminant analysis (OPLS-DA). The unsupervised PCA was implemented to demonstrate the distribution of origin data and general separation. The supervised OPLS-DA was performed to obtain maximal covariance between the measured data and the response variable and validated by 7-fold cross-validation and 200 permutation tests. Kyoto Encyclopedia of Genes and Genomes (KEGG, http://www.genome.jp/kegg/) was utilized to search for the metabolite pathways. MetaboAnalyst, which is a free and web-based tool that uses high-quality KEGG metabolic pathway as the backend knowledge base, was used for the pathway analysis (http://www.metaboanalyst.ca). SIMCA-P+ 14.1software package (Umetrics, Umea, Sweden) was utilized for statistical analysis of the normalized integral values to determine significant differences between metabolic changes. The correlations of certain metabolites with indices of serum hormone, body composition and exercise performance were evaluated with Spearman’s correlation coefficient. All experimental data were expressed as means ± SD, and the data were analyzed by Student’s t-test at significance levels *p* < 0.05. The changes of metabolites with variable importance in projection (VIP) value > 1.5 in the OPLS-DA model and values *p* < 0.01 in the Student’s t- tests were considered to be significantly different.

## Results

### The changes in body weight and the blood biochemical indicators of the studied subjects before and after marathon

All of the studied subjects completed the Marathon match with an average result of 160 min. and 56 s. The body weight was slightly decreased but was not significantly different from that before the exercise. The levels of serum biochemical indicators, CK, urea and cortisol, were all significantly elevated (*p* < 0.01), whereas the level of testosterone was significantly reduced (*p* < 0.01). The level of CRP was significantly increased (*p* < 0.05), and homocysteine was increased but not significantly (Table 1). The results indicate that all the studied subjects were in the state of fatigue after the match.

**Table 1:** Changes of body weight and blood biochemical indexes before- and after-marathon

Indexes	Before	After	*P*-value
Body weight (kg)	61.17 ± 8.27	60.69 ± 8.28	*P* > 0.05
Creatine kinase (IU)	352.39 ± 159.23	543.22 ± 284.16	*P* < 0.01
Isoenzyme (CK-MB) (IU)	20.33 ± 5.30	27.89 ± 7.77	*P* < 0.05
Urea (mmol/L)	4.94 ± 0.84	6.71 ± 1.12	*P* < 0.05
C-reactive protein (mg/L)	0.61 ± 0.21	0.78 ± 0.28	*P* < 0.05
Homocysteine (μmol/L)	14.96 ± 3.45	18.18 ± 9.19	*P* > 0.05
Testosterone (nmol/L)	14.81 ± 4.95	7.36 ± 4.08	*P* < 0.01
Cortisol (nmol/L)	411.56 ± 108.27	773.50 ± 92.01	*P* < 0.01

### Changes of metabolic profiles before and after marathon

**Fig. 1:**
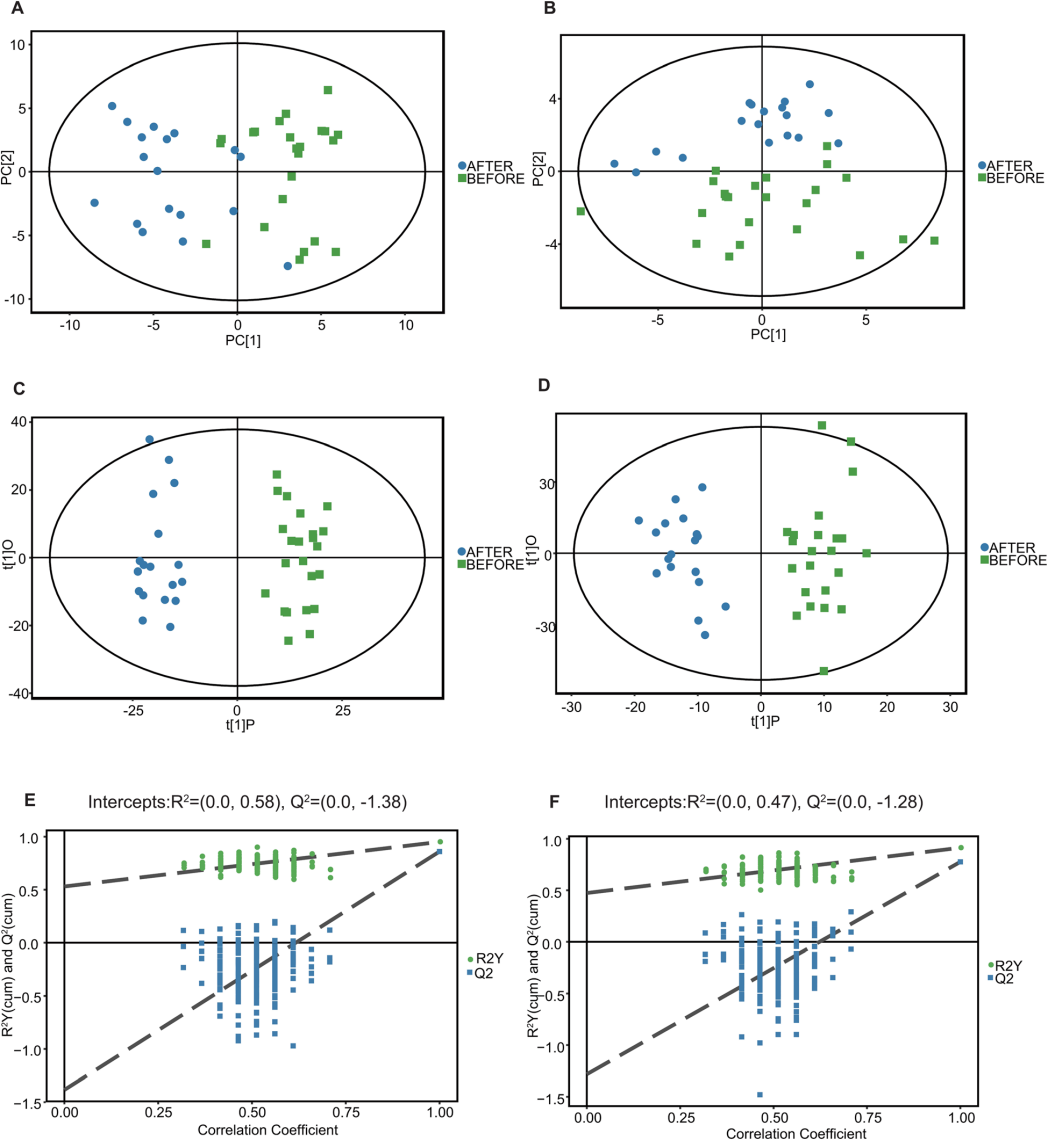
Identification of discriminating biomarkers by metabolomics analysis. The PCA (**a**) and OPLS-DA (**c**) score plot are in negative ion mode. PCA (**b**) and OPLS-DA (**d**) score plot are in positive ion mode following marathon. Validation plot of 200 permutation tests for OPLS-DA model is built for negative (**e**) and positive ion mode (**f**) before and after marathon. The slope of R2 is greater than 0 and the intercept of Q2 on Y axis is less than 0.05, indicating a valid model

The PCA differentiation in the serum profiles (Fig. 1a and b) of the athletes before and after the Marathon match was clearly defined. In order to further examine the metabolic changes, supervised multivariate statistical analysis was used. OPLS-DA model was established to compare two groups’ serum samples. R2 (cum) and Q2 (cum) represented the interpretability and predictability of models, respectively. R2 (cum) and Q2 (cum) were respectively of 0.951 and 0.860 in negative ion mode and 0.913 and 0.776 in positive ion mode, indicating that both models were valid. The scattered shape and color of Fig. 1 represent disparate studied groups. As illustrated in Fig. 1, according to the results of principle component analysis (PCA) and the orthogonal partial least squares discriminant analysis (OPLS-DA) plot scores, it is obvious that the athletes belonging with disparate groups, i.e. before- and after-Marathon match groups, were in different locations. The differences were significant and, based on Hotellings T-squared ellipse analysis, were within 95% confidence interval. In addition, permutation tests were carried out to prevent overfit of the models (Fig. 1 e and f). The abscissa represents the correlation between the random group and the original group, and the ordinate represents the scores of R2 and Q2. The slope of R2 was greater than 0, and the intercept of Q2 on Y axis was less than 0.05, showing that the model had good predictability and did not overfit.

**Fig. 2:**
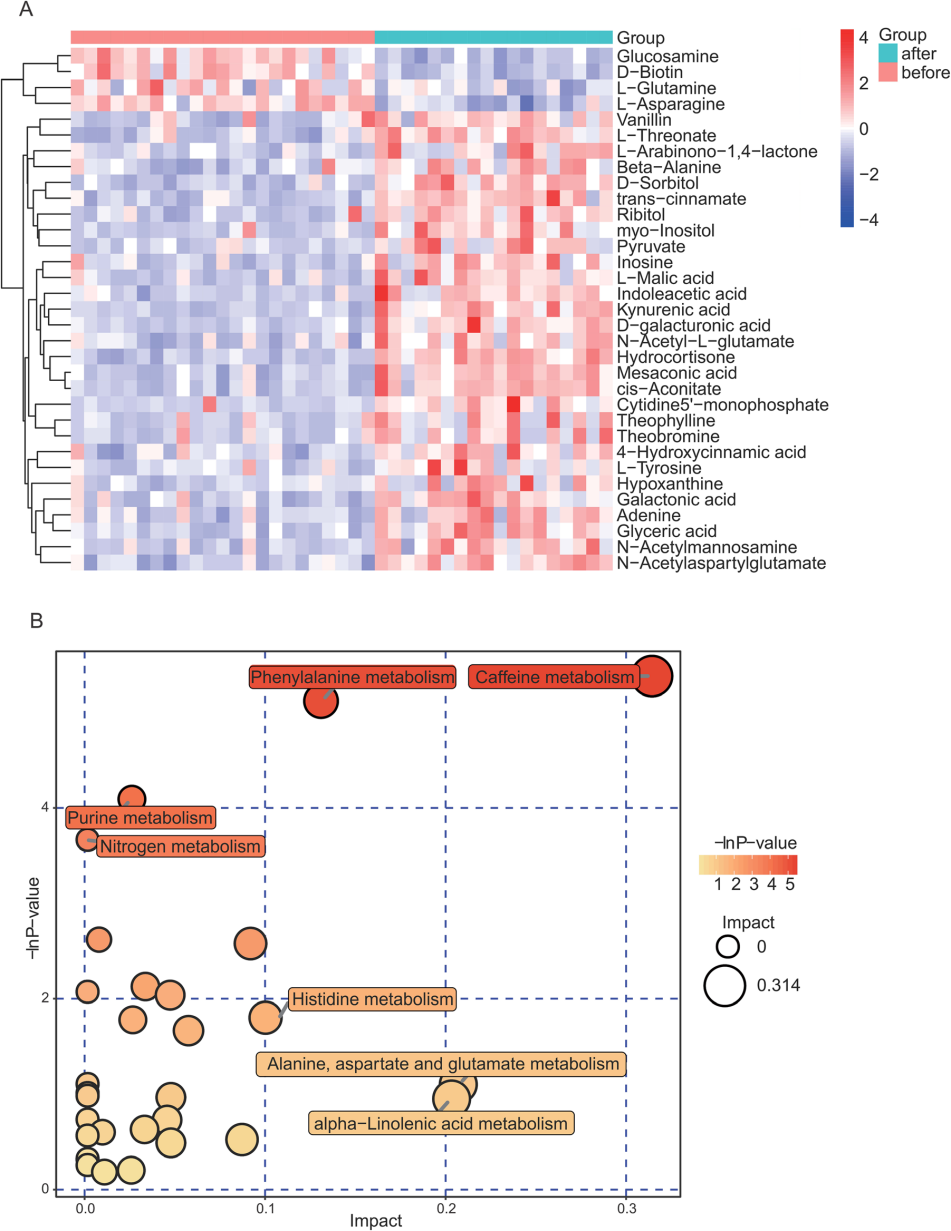
Summary of pathway analyses based on metabolomics data. **a** Heatmap clearly shows the relationship in potential biomarkers between before- and after-marathon serum samples. The Euclidean distance matrix for the quantitative value of the differential metabolites, and cluster the differential metabolites with the complete linkage method. The abscissa represents different experimental groups, the ordinate represents the comparison of the metabolites among different groups, and the color blocks at different positions represent the relative expression of the corresponding metabolites. **b** Bubble plot displays the increasing impact of the enrichment analysis of metabolic pathways, with key bubbles reflecting that the metabolic pathways were significantly altered by marathon

The goal of current study was to obtain a comprehensive view of the altered human serum metabolome following marathon. For UHPLC-Q-TOF/MS, a total of 50 different metabolites were selected based on variable importance in the projection (VIP) values greater than 1.5 and p -values less than 0.01.We then mapped these different metabolites into their biochemical pathways through metabolic enrichment and pathway analyses based on the KEGG database and MetaboAnalyst. According to the results of UHPLC-Q-TOF/MS and GC-TOF/MS analyses, as shown in Fig. 2, the significantly altered pathways were those of caffeine metabolism, alanine, aspartate and glutamate metabolism, pyrimidine metabolism, purine metabolism, and nitrogen metabolism. Table 2 shows the detailed results of the pathway analyses.

**Table 2:** The significant variations in serum metabolite indexes before- and after-marathon groups

Pathway	Metabolite name (pubchem ID)	before	after	*p*-value
Ascorbate and aldarate metabolism	L-Threonate(983)	1.5382 ± 0.2986	2.1988 ± 0.3328	5.79 × 10^− 8^
	myo-Inositol(2148)	0.4216 ± 0.1516	0.7572 ± 0.2611	5.08 × 10^− 5^
	D-galacturonic acid(2578)	0.09 95 ± 0.0190	0.1950 ± 0.0649	7.66 × 10^−6^
	L-Arabinono-1,4-lactone(6329)	0.1846 ± 0.0616	0.3516 ± 0.1631	0.0005
Glyoxylate and dicarboxylate metabolism	Glyceric acid(378)	0.2275 ± 0.0437	0.3689 ± 0.0944	5.69 × 10^−6^
	Mesaconic acid(812)	0.0475 ± 0.0082	0.0759 ± 0.0133	1.47 × 10^− 6^
	L-Malic acid*(927)*	0.0756 ± 0.0221	0.1285 ± 0.0408	4.22 × 10^− 5^
	cis-Aconitate(1987)	0.0242 ± 0.004	0.0399 ± 0.0078	4.56 × 10^− 8^
Phenylalanine metabolism	trans-cinnamate(1311)	0.2978 ± 0.0600	0.4957 ± 0.1099	3.41 × 10^− 7^
	Vanillin(1396)	0.1815 ± 0.1291	0.3046 ± 0.0986	0.0018
	4-Hydroxycinnamic acid(1726)	0.2163 ± 0.0796	0.3401 ± 0.1022	9.07 × 10^− 5^
Carbon metabolism	Glyceric acid(378)	0.2275 ± 0.0437	0.3689 ± 0.0944	5.70 × 10^−6^
	Mesaconic acid(812)	0.0475 ± 0.0082	0.0759 ± 0.0133	1.47 × 10^−8^
	L-Malic acid*(927)*	0.0756 ± 0.0221	0.1285 ± 0.0408	4.22 × 10^− 5^
ABC transporters	myo-Inositol(2148)	0.4216 ± 0.1516	0.7572 ± 0.2611	5.08 × 10^−5^
	D-Sorbitol(2225)	0.4259 ± 0.0923	0.2469 ± 0.0530	9.56 × 10^−8^
	D-galacturonic acid(2578)	0.0995 ± 0.0190	0.1950 ± 0.0649	7.66 × 10^− 6^
	D-Biotin(5747)	1.4304 ± 0.3142	0.8752 ± 0.1676	0.0007
Citrate cycle (TCA cycle)	Pyruvate(182)	0.3757 ± 0.1574	0.8178 ± 0.4326	0.0005
	L-Malic acid*(927)*	0.0756 ± 0.0221	0.1285 ± 0.0408	4.22 × 10^−5^
	cis-Aconitate(1987)	0.0242 ± 0.004	0.0399 ± 0.0078	4.56 × 10^−6^
Pentose and glucuronate interconversions	Ribitol(1400)	0.0573 ± 0.0158	0.0819 ± 0.0152	1.09 × 10^−5^
	D-galacturonic acid(2578)	0.0995 ± 0.0190	0.1950 ± 0.0649	7.66 × 10^−6^
Galactose metabolism	myo-Inositol(2148)	0.4216 ± 0.1516	0.7572 ± 0.2611	5.08 × 10^−5^
	D-Sorbitol(2225)	0.2469 ± 0.0530	0.4259 ± 0.0923	9.56 × 10^−8^
	Galactonic acid(2637)	0.1232 ± 0.0333	0.1895 ± 0.0447	3.05 × 10^−6^
Alanine aspartate and glutamate metabolism	L-Asparagine(879)	0.1203 ± 0.0102	0.0974 ± 0.0114	4.27 × 10^−8^
	N-Acetylaspartylglutamate(6623)	0.0052 ± 0.0009	0.0075 ± 0.0011	3.18 × 10^−9^
Amino sugar and nucleotide suger metabolism	D-galacturonic acid(2578)	0.0995 ± 0.0190	0.1950 ± 0.0649	7.66 × 10^−6^
	Glucosamine(2809)	0.1950 ± 0.0319	0.1284 ± 0.0208	2.68 × 10^−9^
	N-Acetylmannosamine(2874)	0.0279 ± 0.0024	0.0341 ± 0.0048	3.45 × 10^−5^
2-Oxocarboxylic acid metabolism	cis-Aconitate(1987)	0.0242 ± 0.004	0.0399 ± 0.0078	4.56 × 10^−8^
	N-Acetyl-L-glutamate(2440)	0.0158 ± 0.0025	0.0206 ± 0.0033	3.50 × 10^−6^
Central carbon metabolism in cancer	L-Asparagine(879)	0.1203 ± 0.0102	0.0974 ± 0.0114	4.27 × 10^−8^
	L-Malic acid*(927)*	0.0756 ± 0.0221	0.1285 ± 0.0408	4.22 × 10^−5^
Pentose phosphate pathway	Pyruvate(182)	0.3757 ± 0.1574	0.8178 ± 0.4326	0.0005
	Glyceric acid(378)	0.2275 ± 0.0437	0.3689 ± 0.0944	5.70 × 10^−6^
Pyrimidine metabolism	Beta-Alanine(2102)	0.1499 ± 0.0676	0.2418 ± 0.0799	0.0003
	Cytidine5’-monophosphate(7878)	0.0597 ± 0.0470	0.1394 ± 0.0870	0.0018
Purine metabolism	Adenine(949)	0.0192 ± 0.0060	0.0347 ± 0.0087	4.22 × 10^−5^
	Hypoxanthine(984)	1.0917 ± 0.2040	1.7128 ± 0.6085	0.0005
	L-Glutamine(1262)	3.7482 ± 0.5189	3.4252 ± 0.4635	0.0029
	Inosine(5198)	0.4593 ± 0.3814	0.2995 ± 0.1637	9.31 × 10^− 5^
Caffeine metabolism	Theophylline(2152)	0.5386 ± 0.5401	1.0722 ± 0.7971	0.0041
	Theobromine(2187)	0.0432 ± 0.0463	0.0544 ± 0.0465	0.0025
**Steroid hormone biosynthesis**	**Hydrocortisone (10493)**	**0.0927 ± 0.0441**	**0.1241 ± 0.0279**	**0.0017**
Phenylalanine metabolism	4-Hydroxycinnamic acid(1726)	0.2163 ± 0.0796	0.3401 ± 0.1022	9.07 × 10^−5^
	L-Tyrosine(2870)	0.0172 ± 0.0038	0.0198 ± 0.0031	0.0001

### Correlations between the serum metabolic changes and exercise performance

**Fig. 3:**
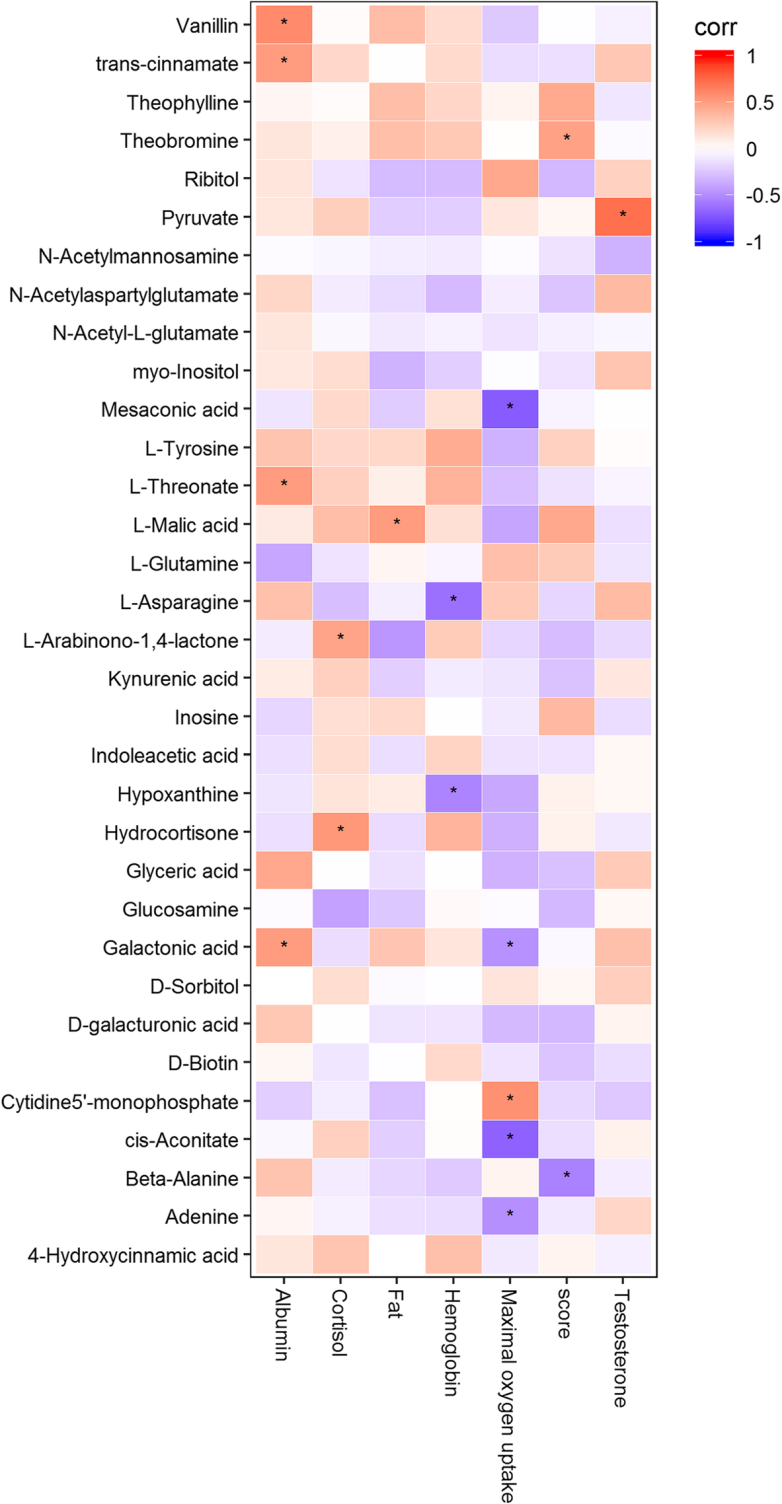
Correlations among exercise performance, fat%, VO_2_max, hemoglobin, and the serum metabolomic indicators

The following results were evaluated according to the score of Marathon achieved by the athletes; the shorter the time, the better the score. The results showed that serum β-alanine was negatively correlated with the score, whereas theobromine was positively correlated with the score (Fig. 3). Additionally, the indicator that reflects the exercise performance, VO_2_max, was negatively correlated with serum cis-aconitate (*r* = − 0.679, *p* < 0.05), galactonic acid (*r* = − 0.474, *p* < 0.05), mesaconic acid (*r* = − 0.708, *p* < 0.05), but positively correlated with cytidine5′ − monophosphate (*r* = 0.562, *p* < 0.05). On the other hand, hemoglobin is often used to evaluate the exercise endurance and functional status. We found that both L-asparagine (*r* = − 0.618, *p* < 0.05) and hypoxanthine (*r* = − 0.530, *p* < 0.05) were negatively correlated with hemoglobin. Generally, for athletes who endure exercise, the less the body fat they have, the better their exercise performance. Our results showed the positive correlation between L-malic acid and the body fat.

### Correlations between serum metabolome and testosterone and cortisol

**Fig. 4:**
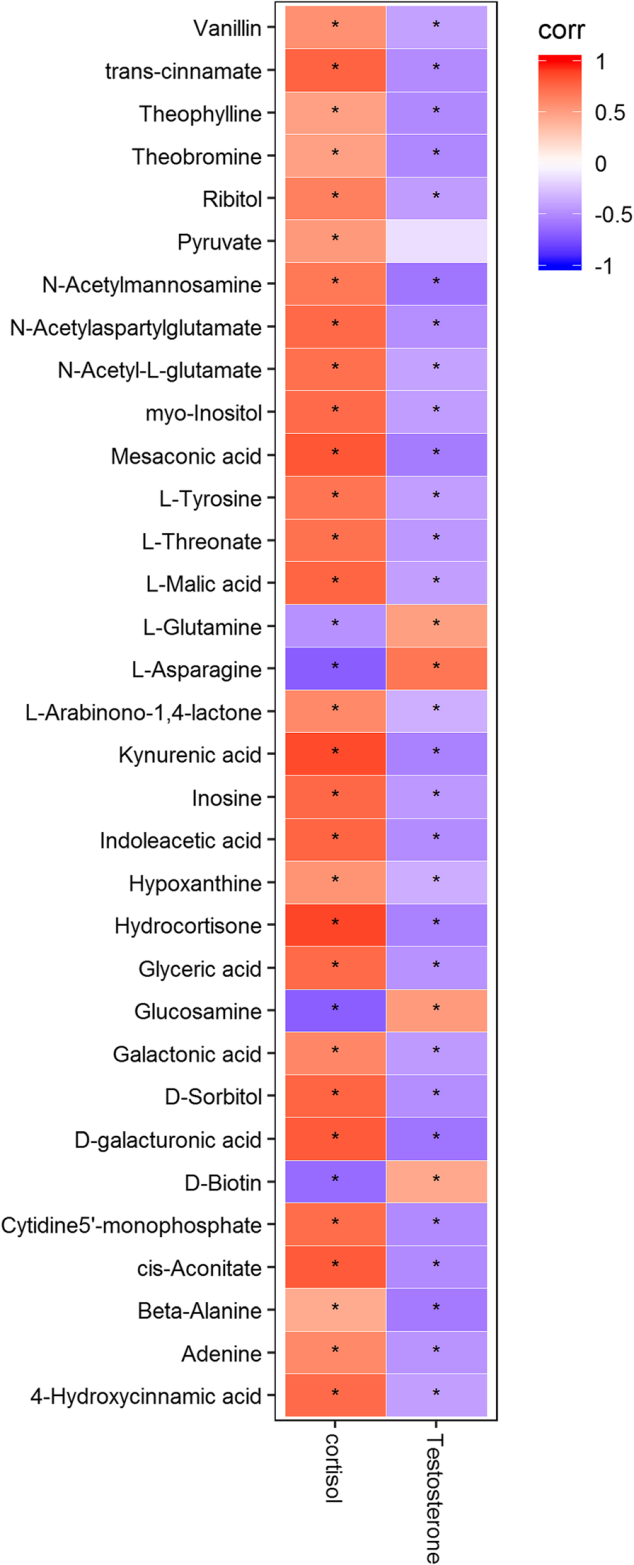
Correlations between serum testosterone and cortisol and metabolomic indicators

Cortisol and testosterone are ideal indicators to reflect athlete’s physical condition and exercise load. Via analyzing the serum levels of cortisol and testosterone before and after exercise, we found that serum testosterone was positively correlated with L-asparagine, L-glutamine, glucosamine, and D-biotin, but negatively correlated with mesaconic acid, kynurenic acid, indoleacetic acid, hydrocortisone, and D-galacturonic acid (Fig. 4). However, as to the indicators of the athletes before exercise, only serum testosterone was positively correlated with pyruvic acid. Additionally, according to the combined data in Fig. 3 and Fig. 4, cortisol was positively correlated with serum hydrocortisone (*r* = 0.536, *p* < 0.05) and L-arabinono-1,4-lactone (*r* = 0.469, *p* < 0.01).

## Discussion

In the current study, all the athletes completed the Marathon match within 3 h with an average score of 160 min and 58 s. Moreover, the body weight of athletes did not change significantly before and after marathon, which was likely due to fluid supplementation during the match.

Urea is the end product of protein metabolism, and prolonged exercises have been shown to cause increased urea concentration in the blood ([9]). Compared to those before exercise, both serum urea and CK elevated significantly. Cortisol stimulates catabolic processes, and cortisol response is enhanced in prolonged exercise. However, estosterone has the opposite effects in exercise [19]. While serum cortisol elevated significantly as well, testosterone decreased significantly. These results indicated that the athletes were in the state of fatigue [20]. In the aspect of evaluating exercise fatigue, Shin demonstrated that the serum urea, CK and other biochemical indicators of the athletes who completed marathon match, were all significantly elevated [21]. Moreover, the athletes subjectively felt fatigue. Additionally, we showed in the current studies that CRP was significantly increased following the match. CRP is among a number of so-called acute proteins that are sharply increased following infections or injuries [22].

Bernat-Adell et al. [23] have discovered that within 24 and 48 h following marathon, athletes’ serum CRP levels were significantly higher than those before exercise. Among the other biochemical indicators, we also found that homocysteine tended to be elevated following the exercise.

Marathon running usually takes about 3 h. It belongs with the aerobic endurance sport, whose energy is provided by the aerobic metabolism of carbohydrates and lipids [24]. The reserve of carbohydrates and the capability of metabolizing the carbohydrates of body are important factors that determine the running score [24]. Our data show that the tricarboxylic acid cycle is intensified, reflected by the elevation of pyruvic acid, malic acid, aconitate, etc. This is confirmed by the accumulation of various TCA cycle intermediates such as α-ketoglutaric acid, succinic acid, citric acid, fumaric acid, and malic acid [25]. The metabolites of the metabolic pathways of other carbohydrates, such as glycerol, inositol, fructose, sorbitol, and galactic acid that are related to lactose, were significantly increased following the exercise (*p* < 0.001), so were acetaldehyde and the metabolites that are related to the metabolic pathway of dicarboxylic acid, as indicated by the elevation of glyceric acid, fumaric acid, malic acid, and aconitic acid. These results confirm again that the TCA cycle is an important pathway that provides the energy for the long-term exercise.

During the long-term exercise, the mobilization of fatty acid as an energy source is proportionally increased, compensating the relative insufficiency of the reserve of carbohydrates. The extent of lipolysis is reflected by the elevation of the levels of serum glycerol and glyceric acid. Like lipolysis, the metabolism of serum caffeine was also enhanced. In the current study, the after-exercise elevation of both serum glycerol and glyceric acid were significantly higher than that of the before-exercise, indicating the proportion of energy provision by the fatty acid was increased. Kujala et al. [19] and Lewis et al. [26] found that, as the energy resource, exercise activated lipolysis and increased the serum levels of glycerol, monopalmitin and various free fatty acids (lauric acid, palmitic acid, palmitoleic acid and 11-eicosenoic acid). Additionally, accumulated 3-hydroxy acids (β-hydroxyhexanoic acid) and 3-keto acids (β-hydroxy-α, β-didehydrosebacic acid) indicated a saturated β-oxidation pathway [27]. Most amino acids are catabolized into TCA cycle substrates via propionyl-CoA, succinyl-CoA, pyruvic acid or acetyl-CoA, depending on the specific amino acid. Reduced concentrations of amino acids (serine, L-valine) were detected in the post-marathon serum, which indicated amino acid catabolism during the Marathon.

During the long-term exercise, to compensate the insufficient energy supply by carbohydrates, energy supply via proteolysis is elevated. However, because of the differences in the capability to provide the energy, proteolysis may lead to the imbalanced protein metabolism. We found that serum alanine, phenylalanine and N-acetylglutamic acid were significantly elevated following the exercise. Normally, because of the elevated energy metabolism, exercise induces the increased deamination of amino acids, leading to the increased metabolism of glucose-alanine cycle [28].

In addition to the energy-supplying pathways, the metabolism of glucosamine was also changed following marathon. The levels of D-galacturonic acid and N-acetylneur aminic acid were significantly elevated whereas those of glucosamine and N-acetyl-glusanine were significantly decreased. As glucosamine is an important nutrient substance that maintains the structure of joints and cartilage, previous investigation showed that the level of glucosamine was reduced in the process of aging, particularly when the function of joint was degenerated [29, 30]. The studies conducted by Henrotin et al. [31] also found that long-term exercise could lead to inflammation of the joint, accompanied by the reduction in the level of glucosamine. Supplementation of exogenous glucosamine could alleviate and treat the joint inflammation. Together with our data, supplementing glucosamine can benefit the long-distance athletes to maintain adequate nutrient status. The major food resource of glucosamine is from the shelled seafood. Marathon trainees are recommended to supplement this type of nutrient.

An interesting observation is that caffeine metabolism was intensified, indicated by the significant increase in theophylline and theobromine. The elevated caffeine, theophylline and theobromine may mobilize fatty acid to provide energy. Potgieter et al. [32] found that supplementation of exogenous caffeine increased the activity of hormone-sensitive lipases and serum levels of glycerides, leading to the simulation of lipolysis. On the other hand, the elevation in theophylline and theobromine may be related to euphoria. Paraxanthine (1,7- paraxanthine), an euphoria-related substance, can accelerate lipolysis, leading to an increase in the serum levels of triglyceride and free fatty acids. Theobromine can dilate blood vessels and increase urine output, whereas theophylline may relax the smooth muscle of bronchus. These functions are related to central nervous system excitation, muscle tremble and euphoria. The craziness for marathon, even to the extent of addiction, among a large number of marathon enthusiasts is probably associated with the promotion of the metabolism of caffeine as a result of long-term exercise, further showing that the craziness for marathon is associated with the metabolism of caffeine in the body.

Long-term training in exercise leaves profound marks in both the epigenetic and metabolomics profiles of athletes. The other goal of our studies was to investigate the relationship between the marathon runners and their metabolomics. VO_2_max is an important indicator that reflects the endurance of athletes. Generally, the VO_2_max of marathon runners is relatively higher [5]. Our data showed that serum cis − aconitate, galactonic acid and mesaconic acid were negatively correlated with VO_2_max whereas cytidine 5’ − monophosphate was positively correlated with VO2max. Both cis − aconitate and galactose are intermediates of energy metabolism, and cis − aconitate is the intermediate of citric acid cycle. When aerobic capabilities are increased, that is when VO_2_max is increased, these serum metabolites are decreased as a result of being utilized. Lewis et al. [26] found that the serum level of triglyceride was closely related to lipid metabolism and aerobic capabilities.

Hemoglobin often indicates the physical abilities and performance capacities. We found that both L-asparagine (*r* = − 0.618, *p* < 0.05) and hypoxanthine (*r* = − 0.530, *p* < 0.05) were negatively correlated with hemoglobin. Hypoxanthine is a metabolic product of purine metabolism. When skeletal muscle cells are under the condition of ischemia and hypoxia or when ATP catabolism exceeds its anabolism, particularly when the energy status is negatively balanced, the serum level of hypoxanthine is elevated [33]. Therefore, hypoxanthine may be regarded as an important metabolic indicator to reflect the level of hemoglobin and metabolic state of the energy in the body.

Serum cortisol and testosterone are important indicators of functional status and exercise load. Cortisol is produced primarily under stress, particularly under strenuous exercise. Under these conditions, the body consumes a large number of energy-producing substances. These substances subsequently activate the hypothalamus-pituitary gland-adrenal gland axis, intensifying cortisol production [34]. Cortisol was positively correlated with serum hydrocortisone (*r* = 0.536, *p* < 0.05) and L-arabinono-1,4-lactone (*r* = 0.469, *p* < 0.01).

Hydrocortisone is a metabolic product of steroid hormone biosynthesis. Peake et al. [35] showed that steroid hormone biosynthesis was not impacted by any specific types of exercise. However, intensified exercise often increases its metabolic product hydrocortisone and also further promotes catabolism. Moderate to high intensity exercise that exceeds 60% of VO2max causes intensity-dependent elevation in blood cortisol levels [36, 37].

The changes in the level of testosterone reflect the physical abilities in the resting state. Exercise often reduces the level. Our data showed that testosterone was positively related only to the pre-exercise but not the after-exercise metabolic product pyruvic acid. Pyruvic acid is an intermediate product of the metabolism of carbohydrates, lipids and amino acids. Pyruvic acid is also a pivotal intermediate of metabolic pathways in a cell. It is linked to both TCA cycle and hexose diphosphate pathway, two important biochemical metabolic cyclic pathways. Pyruvic acid is the center that links glycolysis, lactic acid, acetyl-CoA, oxaloacetic acid, malic acid, and various amino acids [38]. It is the intermediate that plays a pivotal role in the energy metabolism. Acute venous injection of pyruvic acid was beneficial to the performance of exercise in the rats [39]. Moreover, animal studies showed that the level of glycogen was increased and the utilization of carbohydrates by the skeletal muscle was improved in the rats that received long-term supplementation of pyruvic acid [40]. Our data also showed that in the resting state, testosterone and pyruvic acid were positively correlated in the marathon runners, which could reflect the physical condition of the athletes.

## Conclusion remarks

In summary, our studies found that the exercise promoted the catabolism of carbohydrates, lipids and amino acids, and potentiated the TCA cycle. These metabolic changes increase energy supply for the runners. Our results also showed that the serum glucosamine and N-acetyl-glusanine of runners were significantly reduced after the match. The results provide evidence that supplementation of glucosamine restores the glucosamine in the athletes. Moreover, the significant elevation in caffeine metabolites, theophylline and theobromine, following the exercise, provides evidence for the observations of not only that the energy supply can be promoted via lipid metabolism but also the addiction to exercise.

Our studies found that serum cortisol could accurately reflect working load and physical abilities. Moreover, it was positively correlated with the serum metabolites hydrocortisone and L-arabinono-1,4-lactone. Our studies also discovered that serum metabolites (cis-aconitate, galactonic acid and mesaconicacid) are closely related to VO_2_max, but further research is needed. Additionally, hypoxanthine could be regarded as an important metabolomic indicator that reflected the level of hemoglobin and the energy state in the body.

## Data Availability

Current research is part of a larger research project, and some of the results are used only for reference by athletes and researchers. Considering this, the datasets generated from this investigation are not publically available, but can be acquired from the corresponding author on reasonable request.
