# Correction to: Runners’ metabolomic changes following marathon

**DOI:** 10.1186/s12986-020-00468-6

**Published:** 2020-07-07

**Authors:** Rengfei Shi, Jin Zhang, Biqing Fang, Xiangyang Tian, Yu Feng, Zepeng Cheng, Zhongyu Fu, Jingjing Zhang, Jiaxi Wu

**Affiliations:** 1 School of Kinesiology, Shanghai University of Sport, 188 Hengren Road, Yangpu District, Shanghai, 200438 China; 2 Central Laboratories, Xuhui Central Hospital, Shanghai Clinical Research Center, Chinese Academy of Sciences, 966 Huaihai Middle Road, Shanghai, 200031 China

**Correction to: Nutr Metab (2020) 17:19**

**https://doi.org/10.1186/s12986-020-00436-0**

Following publication of the original article [1], the authors identified a missing reference.

Stander, Z., Luies, L., Mienie, L.J. et al. The altered human serum metabolome induced by a marathon. Metabolomics 14, 150 (2018).

https://doi.org/10.1007/s11306-018-1447-4
